# Research assistants’ experiences recruiting patients with psychosis into clinical trials: a qualitative study

**DOI:** 10.1186/s13063-025-08882-y

**Published:** 2025-05-30

**Authors:** Ariane Beckley, Margaret Glogowska, Felicity Waite, Penny Bee, Daniel Freeman

**Affiliations:** 1 Department of Experimental Psychology, University of Oxford, Life and Mind Building, South Parks Road, Oxford, OX1 3RF UK; 2 Nuffield Department of Primary Care Health Sciences, University of Oxford, Oxford, UK; 3 Oxford Health NHS Foundation Trust, Oxford, UK; 4 Division of Nursing, Midwifery and Social Work, University of Manchester, Manchester, England UK

**Keywords:** Psychosis, Trial, Recruitment, Research assistant, Qualitative research

## Abstract

**Objectives:**

Treatments for patients diagnosed with psychosis need to be improved. Clinical trials are an important way of assessing the efficacy of new treatments. However, recruiting patients into trials is challenging. This study sought to better understand the reasons for this from the perspective of research assistants.

**Design:**

A qualitative study underpinned by a critical realist ontology and contextualist epistemology.

**Methods:**

Research assistants who had recruited patients with psychosis into trials, primarily of psychological interventions, were interviewed. Reflexive thematic analysis was used to identify themes.

**Results:**

Overarching themes representing four types of factors influencing recruitment of patients with psychosis into clinical trials were generated: patient, clinical team, research team, and NHS infrastructure. Patients largely wished to take part in trials but needed time to build trust with research assistants. Clinical teams held the power in suggesting patients for trials; therefore, it was essential for research teams to build strong relationships with clinical staff. Research teams recruiting into trials benefited from lived experience expertise, support systems, and institutional knowledge. A key NHS infrastructure factor was that mental health staff had limited time to consider trials for their patients.

**Conclusions:**

Trial participation needs to be made more accessible to patients with psychosis, who often want to take part but lack opportunities. Methods of increasing accessibility could include identifying and addressing barriers to referral from clinical teams, employing multiple recruitment strategies, and flexible appointment formats. Qualitative research with clinical teams and patients will also help in developing the understanding of barriers to recruitment.

**Supplementary Information:**

The online version contains supplementary material available at 10.1186/s13063-025-08882-y.

## Background

Psychosis is typically associated with distress, social exclusion and difficulties studying, working and maintaining relationships [1]. Life expectancy with the most common psychotic disorder, schizophrenia [1], is on average 14.5 years lower than for the general population [2]. This is due to increased mortality from a host of causes—many of which are preventable—including suicide, pneumonia, respiratory disease, diabetes, and cancer [3]. Current pharmacological and psychological treatments for psychosis are insufficiently effective for too many patients [4, 5]. Finding new interventions which work and are safe is therefore crucial.

Clinical trials are the most robust way of testing the effectiveness of new interventions [6]. However, recruiting participants is a challenge [7]; a recent 2022 review of publicly funded UK trials has found that nearly 40% fail to recruit to target [8]. Ineffective recruitment can result in higher costs, a longer duration, and reduced staff morale [9], as well as inconclusive results due to insufficient statistical power [10] and limited generalisability [11]. Trials in secondary care mental health settings face additional complexities: stigma surrounding mental health conditions can deter patients from participating [12], patients’ capacity to consent can fluctuate [13], and trial teams typically rely on clinical teams to refer suitable patients [14].

The small number of studies on the recruitment of patients with psychosis into trials has primarily been studies within trials (SWATs). These have explored the experiences of research and/or clinical staff recruiting into one specific trial. The main barrier that has been identified by these designs is that clinical teams do not always refer patients. Reasons for this include clinicians lacking time [15–17]; fearing that trial participation could cause patients distress [15, 18, 19]; applying additional eligibility criteria beyond those specified in the trial protocol, for example, patients needing to be well engaged with services or likely to adhere to the intervention [15–18]; and limited opportunities for clinical and research teams to liaise [15, 16]. Facilitators to recruiting patients with psychosis into trials that have been identified include research teams actively maintaining good relationships with clinical teams [15, 17–19]; researchers employing soft skills such as flexibility, persistence and professionalism [15]; and clinicians having a positive view of the intervention [16, 18].

Studies on the perspective of patients are very limited, although one study explored the views of cannabis users with first episode psychosis (FEP) on the barriers and facilitators to participation in a randomised controlled trial (RCT) for substance abuse [20]. Facilitators were personal benefits (access to therapy, greater self-knowledge, social contact with researchers), helping others, the assurance of confidentiality, and encouragement from significant others. Barriers were related to the research procedures and included the difficulty and length of assessments, and finding discussing past experiences distressing.

The primary recruiters into clinical trials are research assistants (RAs). The research assistant role is usually a fixed-term graduate entry position that is junior within a research team and linked to a particular study. Key responsibilities typically include recruiting and consenting participants, liaising with clinical teams, administering assessments, data entry, and administrative duties. RAs are therefore particularly well placed to describe the difficulties encountered during recruitment and suggest ways of potentially improving recruitment practices. This study aims to add to existing research on recruiting patients with psychosis into clinical trials by establishing an understanding of the barriers and facilitators from the perspective of RAs employed on recent randomised controlled trials.

## Methods

### Design

A qualitative design was used to conduct an in-depth exploration of RAs’ experiences of recruiting patients with psychosis into clinical trials. The study adopted a critical realist ontological position [21] and a contextual epistemology [22] with RAs perceived as interpreters of the realities of trial recruitment within the local context of the trial(s) they had worked on and the wider context of the NHS. As the aim was to explore the RAs’ perspectives, the study took an experiential orientation, accepting rather than challenging their accounts.

### Procedure

Participants were recruited by circulating a flyer advertising the study through the researchers’ networks. Trial coordinators and PIs (principal investigators) working on psychosis trials across the UK and known to the researchers were asked to share the flyer with any potentially suitable current or former colleagues. The trial coordinators and PIs were known to have worked on trials testing a range of interventions and to have recruited both remotely and in person, meaning that there would be a variety in recruitment experiences.

The first author responded to those who expressed interest in taking part in the study. Prospective participants were given at least 24 h to read the participant information sheet (PIS). The PIS described the first author’s background in trial coordination and interest in improving trial recruitment, explained that the study was being conducted as part of a Master’s degree in Clinical Research at the University of Manchester, and detailed what taking part would involve. A remote interview was arranged with those who agreed to participate and met the inclusion criteria.

Inclusion criteria were: aged 18 years or above; works or has previously worked as a research assistant or in an equivalent role; recruited participants with psychosis into at least one clinical trial in the last three years; able to take part in a remote interview in English about their experiences of trial recruitment and be located in the UK at the time of the interview; and willing and able to give informed consent for participation in the study.

The planned sample size was based on the principle of ‘information power’ [23]. The sample size was reviewed during the study, and recruitment stopped at 15 participants as the sample was judged to possess sufficient explanatory power to answer the research question.

Data were collected by semi-structured interview as this is suitable for exploring participants’ accounts of phenomena in detail. The first author, who had training in and experience of qualitative interviewing, conducted the interviews. The interviews were informed by a topic guide, which covered the RA’s background in recruiting patients with psychosis into clinical trials, recruitment processes they had followed, liaison with clinical teams and patients, recruitment challenges, and ways of improving recruitment (see supplementary materials). The topic guide was tested for clarity with the first two participants, and minor changes were made to the order of topics as indicated. Participants were also given the opportunity to raise issues of importance to them.

The interviews took place remotely between February and May 2022 with each participant interviewed once. The interviewer was located at home or in a private office with no one else present. Before the interview began, verbal informed consent was obtained. Basic demographic information and summary details of the RA’s experience recruiting participants with psychosis into trials were collected for each RA via a form. The interviews were audio recorded using an encrypted Dictaphone. The researcher took notes during the interviews and recorded reflections afterwards in a reflexive journal. The recordings were transcribed verbatim by a professional transcription company. The transcripts were checked for accuracy and de-identified.

### Positionality statement

The first author, who conducted the interviews, is female and her ethnicity is White British. She has a degree in Psychology and works as a trial coordinator in a clinical psychology research team with a strong patient-centred ethos. Prior to conducting this study, she worked on two clinical trials that did not recruit to target, which provoked her interest in researching trial recruitment.

### Data analysis

Data were analysed by hand and with NVivo 1 using reflexive thematic analysis (TA) [22]. Reflexive TA entails the identification of themes (recurring concepts or ideas) in the data and emphasises the active role of the researcher in interpreting them. The researcher’s ability to recognise how their personal and professional experiences, views, and values inform the conduct of the research and the relationship between researcher and participant is a strength in reflexive TA [22].

Data analysis began after the first few interviews so early themes identified could be followed up in subsequent interviews. The first author was the only coder, consistent with reflexive TA’s foregrounding of subjectivity [22]. The analysis followed the six-phase procedure outlined by Braun and Clarke [22]: building familiarity with the data by checking the transcripts against the recordings and reading the transcripts repeatedly; coding each transcript by highlighting content relevant to the research question; grouping codes with a shared meaning together to begin to form themes; comparing the themes to the transcripts to ensure credibility and elaborating and rejecting themes as necessary; clarifying and naming the themes; writing the report.

One thousand five hundred sixteen references were coded. These initial codes were organised into 22 themes by gathering together those which shared a common idea. The themes were reconceptualised and relabelled to make them more inclusive and coherent, reducing the number to the four themes and 10 sub-themes reported. As the analysis progressed, it became apparent that the barrier/facilitator binary originally envisaged was a reductive framework, so the focus was broadened to considering the factors influencing the recruitment of patients with psychosis into clinical trials instead. In order to enhance the credibility and dependability of the analysis [24], another researcher reviewed coding samples. This helped to foster reflexivity and identify any oversights; for example, a question about the emotional aspects of trial recruitment was added following the coding review. The construction and interpretation of the themes was discussed and refined with the supervisory team during regular supervision sessions. Further details on how methodological rigour was considered are included in the supplementary materials.

### Ethical considerations

Because RAs were recruited via the researchers’ personal networks and the first author is a trial coordinator in a clinical research team carrying out trials involving patients with psychosis, some interviewees were already known to the researcher professionally. To ensure current colleagues or former supervisees did not feel under pressure to agree to take part in the study, it was emphasised to all potential participants that they had the freedom to decide whether to participate and if they did agree to take part, that they could change their mind. A distress protocol based on Draucker et al.’s [25] template for interviews on sensitive topics was in place in case of participant distress. Participants were reassured that their data would be kept confidential, except in the case of a disclosure of professional misconduct.

## Results

### Participant characteristics

Of the 19 individuals who expressed an interest in participating, three did not meet the inclusion criteria regarding recruitment experience and one who was eligible ceased to respond to communications. The remaining 15 were interviewed. Participant characteristics are shown in Table 1.

**Table 1:** Participant characteristics (*N* = 15)

Demographic characteristic	No. of research assistants
Age (years)	
20–24	4
25–29	9
30–34	2
Gender	
Female	12
Male	2
Non-binary	1
Ethnicity	
White British	12
White Scottish	1
Pakistani	1
Pakistani British	1
Length of time in recruiting role	
≤ 1 year	8
> 1 year	7
No. trials recruited patients with psychosis into	
1	9
2	2
3	2
≥ 4	2

All participants had recruited patients with psychosis into randomised controlled trials (RCTs) of psychological interventions. In addition, one participant had recruited into RCTs of other nonpharmacological interventions and two into RCTs involving pharmacological interventions. 10 had recruited remotely, either exclusively or in addition to face-to-face.

Interviews lasted between 33 and 75 min.

### Themes

Overarching themes representing four types of factors were generated by the thematic analysis: patient, clinical team, research team, and NHS infrastructure, as shown in Table 2. These themes capture the key agents in the recruitment process (patients, clinical teams, and research teams) and the wider context (NHS infrastructure) in which they exist.

**Table 2:** Themes and sub-themes

Themes	Sub-themes			
*Patient factors*	Wanting to take part		Building trust	
*Research team factors*	Lived experience expertise	Support system		Institutional knowledge
*Clinical team factors*	Holding the power	Judging suitability		Relationships are the crux
*NHS infrastructure factors*	Systemic issues		Alternative recruitment pathways	

#### Theme 1: Patient factors

There were two sub-themes related to patients: wanting to take part in trials and needing to trust RAs in order to feel comfortable participating.

##### Wanting to take part

RAs reported that patients were typically positive about the prospect of clinical trial participation. Once the RA had reached the stage of making direct contact with the patient, the rest of the recruitment process tended to proceed without difficulty:

Most people […] I think, quite wanted to take part but I think it was the referral numbers from clinicians that was tricky to get. (Interview 5)

RAs reported that patients were primarily motivated to participate by the opportunity to access effective psychological support, which was not otherwise readily available:

[I]t was just this desperation of trying to find something that would work and being really willing to try anything if it helped. […] a lot of people really struggle with medication and psychosis, so the idea of finding more effective psychological therapies as well was something that a lot of people kind of wanted to get involved with. (Interview 6)

Due to long waiting lists in services, trial participation also provided an opportunity to access such support faster:

[T]he wait lists for therapy are long. And to be told that you might be able to be seen earlier, even if you’re not seen earlier, you’d still be paid if you take part in assessments, I think people were happy just to be able to receive support at an earlier stage. (Interview 15)

Despite there being a widespread assumption amongst the RAs that the possibility of randomisation to the control arm would deter patients from participating and clinicians also articulating worries to this effect, as the above quotation indicates, patients were often accepting of the uncertainty. RAs reported that sometimes patients felt that there were sufficient other benefits beyond the possibility of receiving the intervention to justify trial participation:

I think some people didn’t really mind the treatment as usual group ‘cause they were like, ‘oh well, regardless, I’m still meeting with someone, I’m doing questionnaires and getting some money out of it.’ (Interview 12)

For other patients, randomisation was simply not of particular concern:

I think it was almost like they didn’t really care, if that’s like the nicest way to put it? They were just like, ‘yeah, I’m helping out, I could get therapy, or I could not.’ (Interview 11)

The desire to ‘help’ was noted by many RAs, and altruistic motivations were for some patients the main driver for participation:

[A] lot of the time participants were really happy to be part of something sort of wider and something that was going to contribute to helping people who’d experienced things like them… (Interview 7)

##### Building trust

Patients with psychosis can experience paranoia and find it difficult to feel safe around others. The RAs needed to work to establish a sense of trust with each patient for them to be willing to participate in the trial:

[T]he main one [challenge] probably would be building trust with people, because I guess some of the symptoms are sort of, you know, being suspicious of others, or fear of judgement, or fear of, you know, I guess, embarrassing yourself. […] I was very aware that I needed to build those relationships with people, for them to engage throughout the trial. (Interview 9)

Time and patience were needed to successfully establish trust:

I think that going too fast can cause too many… I think it can, for some people it can be a rupture at the very start and you need to be able to build that trust so that people know what’s going on, who you are, why you’re doing it. (Interview 15)

Learning about the individual patient’s preferences and accommodating them helped to foster feelings of safety:

[H]aving that flexibility to meet them [patients] in their home, which might make them feel more safe. But also with some people like not assuming that. […] your going to their home might, might feel quite unsafe for them, so then meeting […] at their base where they go for their normal clinical meetings with their care team, might feel safer. (Interview 5)

Attending joint appointments with care coordinators was a way to build trust with patients for many of the RAs:

[I]t was just having them [the care co-ordinator] there, ‘cause it’s a friendly face. […] I know trust can be like a big deal in like psychosis, so it’s like, ‘okay, they’re there, I trust them.’ It helps kind of knowing you can trust the other person at the same time (Interview 1)

In contrast, one RA felt that for patients from ethnic minority backgrounds, their relationship with their care coordinator could have contributed to their difficulties trusting others:

[H]aving experienced racism from care coordinators, so why would they [patients] then want to engage with a care coordinator to get referral to a trial, or why would they trust a psychologist or a research assistant, or assistant psychologist […] to speak to them about their experiences in that baseline assessment, you know? It just takes, I think, more time and effort to reassure those people and build that connection with groups that, for so long, have just had bad experiences with clinical services. (Interview 13)

For the RAs who had recruited both face-to-face and remotely during the COVID-19 pandemic, the latter was typically viewed as inferior, as interacting online or over the phone made establishing a rapport with both clinicians and patients more difficult. One RA stated:

I think the nature of psychosis and being mistrustful of others and paranoid and unsure of people’s intentions, I’m essentially just the voice at the end of a phone that this person’s meant to like trust that you’re legitimate. (Interview 8)

However, one RA felt there was little difference between the two methods:

from my personal experience of it having been remote, I didn’t really think there were too many issues, or that the people I did recruit in person wasn’t any easier [a] process than someone remote. (Interview 9)

Others had a positive view of remote recruitment. One RA saw being able to offer patients appointments in different formats—on the phone, video call, face-to-face—as an opportunity to empower participants and put them at ease:

I think participants really liked having all those choices and I think it made them feel like they had a sense of control over their own participation… (Interview 4)

Another RA thought that remote recruitment felt more comfortable for participants who were anxious about leaving the house and social interaction:

I think some people found it easier because they were worried about going outside and meeting others, and being over the phone they didn’t have to do that, they just had to pick up the phone and chat to someone. (Interview 7)

#### Theme 2: Research team factors

There were three sub-themes relating to research teams: the value of lived experience; support systems; and institutional knowledge.

##### Lived experience expertise

The RAs described how their research teams incorporated the insights of people with lived experience of psychosis into their recruitment strategies to ensure they reflected patients’ needs and preferences. One RA described how having research team members with lived experience benefitted patients, other research team members, and the research itself:

[T]he participants you’re working with really want to have someone that understands. And I think sort of having a team that have people that understand in the team was so helpful for like group discussions and also just like […] if you just needed to talk to someone, and like share that you were struggling with something, they would be really helpful. But I think as well, it’s just like to frame the research right, to make sure that what you’re asking and doing is not, it’s just yeah in line with them, it’s not going to like offend anyone, and you’re getting at the right problems as well. (Interview 11)

The involvement of people with lived experience was also perceived as enhancing the appeal of the trial to clinical teams and thus increasing referrals by a number of RAs, for example:

I think when people had heard that people with lived experience had been involved in developing the project and thinking about this […] that kind of made people more excited about it and a bit more willing. (Interview 12)

##### Support system

RAs described finding trial recruitment emotionally and practically challenging and relied on support from their research team to manage these challenges. One RA described the sense of solidarity in their team:

I think the thing for me that made it possible was being in a really supportive team. So, like other research assistants and clinical psychologists and researchers in the team that I was based in, like that being a really important support system and working together effectively to recruit (Interview 5)

Supervision from more senior team members and sharing experiences with other research assistants who were able to empathise were highlighted as particularly beneficial when dealing with difficulties:

[I]t [supervision with clinical psychologist] does help you like put into perspective again, like why someone’s not turned up, or like what’s going on for somebody […]. So, I think it definitely helps you to let out your frustrations and then think kind of compassionately towards a person... (Interview 8)

##### Institutional knowledge

RAs are typically only in their role for a relatively brief period of time, with staff often changing during the course of a trial. For this reason, being able to access institutional knowledge—skills, experiences, and expertise acquired by predecessors and other team members—about recruitment was useful. Sometimes this knowledge might be trial-specific and passed on by a particular individual, as described by one RA:

I came in halfway through the trial though because I was taking over from someone who had already been in the trial for a year. So I think they gave me this huge folder of techniques and scripts and [….] things that they’d already discovered were helpful. So I had kind of a good sort of foundation... (Interview 6)

In other cases, recruitment strategies were based on learning acquired during other studies, which was part of the team’s collective memory, as for another RA:

I would say it’s mainly things that they’ve [the research team] found that have worked before, based on previous studies… (Interview 10)

Research teams being part of the local research culture of their organisation and attuned to what other trials are recruiting afforded opportunities to recruit more effectively:

I think it’s leeching off other trials that are around us and have those connections, so that was really helpful in starting us up because we were running alongside a quite prominent research trial that had a lot of connections in the teams […] And the teams would already be quite aware of that trial and how, you know, randomised controlled trials work, which was absolutely brilliant for us because then we wouldn’t be having to explain exactly how research works… (Interview 3)

#### Theme 3: Clinical team factors

There were three sub-themes related to clinical teams: holding the power; judging suitability; and relationships are the crux.

##### Holding the power

Although patients may be keen to take part in clinical trials (theme 1), the first step is for them to be referred to the trial by their clinical team. The RAs perceived the attitudes and behaviours of clinical teams as determining whether trials recruited successfully or not. Some teams were ‘research minded’ (as a number of the RAs put it); valuing research and facilitating it. Often, teams saw that referring patients could have benefits for them, as described here:

[G]enerally, I think, like team managers were really sort of on board and thought it was a really good thing, and I think when they framed it more as, ‘this could be really helpful towards like what we can send someone to do, you know, refer someone […] and then that might help with the waitlist’ (Interview 11)

However, this was not always the case. Many of the RAs shared the view that:

[T]he main barrier [to recruitment] is that some service users are being told, ‘there’s this,’ and some aren’t. And it’s about sort of making sure it’s not the case and that everyone is told who wants to be told about research and given that chance (Interview 10)

One RA described some trials where recruitment had been particularly difficult because the inclusion criteria included symptoms or experiences (in addition to psychosis) that clinical teams did not routinely ask patients about:

So they didn’t know, and then that meant that they couldn’t refer people to the trial and that then meant that the trial under-recruited. (Interview 13)

Referring patients was also perceived by clinical teams as additional work that was not a good use of their limited time:

[S]ome teams may view it as, ‘oh, this is a burden, like we don’t have time for this. You know, service user might not want to take part, we’re not giving them the choice.’ (Interview 14)

In response to this reluctance, RAs saw themselves as using ‘sales skills’ to facilitate buy-in from clinical teams and generate referrals. One RA described this:

[I]t’s holding that excitement for everyone and really trying to deliver that bit, because people get excited with you. When it’s almost like, ‘oh, this is a big favour and I’m asking you to do this,’ that’s not so exciting. Whereas if it’s sort of that this could really be life changing and this could be an amazing new treatment, […] that’s where people kind of really want to get involved. (Interview 6)

Another RA described how they promoted the trial, giving merchandise to clinical teams to keep the trial at the forefront of clinicians’ minds:

[W]e took in sort of promo materials, we took t-shirts, pens, these sticky Post-It notes, and tried to… and mugs […] and make it so that, you know, they [clinical teams] weren’t able to forget what we were promoting. (Interview 9)

##### Judging suitability

Whether a particular patient was referred or not depended on whether their clinical team judged them to be suitable. Determining patient eligibility for a trial was viewed as subjective and therefore not self-evident. As one RA put it:

[S]ome of the exclusion criteria are things that in their nature not completely objective, and they involve discussions with the team (Interview 2)

RAs reported that poor engagement with services, substance abuse, and the inability to leave the house were commonly given as reasons for clinical teams not referring potential participants. However, these were typically not included in the trial eligibility criteria and were often difficulties that the research team were actually used to dealing with:

[A] big thing that I did find come up quite a lot, was that, like, ‘oh, would this person be able to do it, ‘cause they don’t leave their home?’ and, ‘oh would this person be able to do it, because they take drugs, morning, afternoon and evening?’ And […] then we were like, ‘yeah, no, we can actually potentially like look into that…’ And they’d almost, clinicians might have mentally sort of ruled a lot of people out because of their difficulties… (Interview 11)

One RA described proactively asking clinicians why they were not referring patients and then finding solutions to the issues raised:

I find myself being quite assertive, like maybe saying, like, ‘Oh do you have any concerns?’ or, ‘What would put you off referring somebody?’ You know, like, ‘Oh they’re treatment resistant,’ and I’d say, ‘Well it might not surprise you, like kind of, people in [trial name redacted], there’s persistent distressing voices, and like, prescribed antipsychotics. So most of our caseload are on clozapine, so that absolutely would not be an exclusion. In fact, would it be possible to come along to your clozapine clinic?’ And I suppose like kind of, asking what the barriers are, and then just formulating a bit, trying to find a facilitator, or a negotiator, even. (Interview 14)

RAs observed patients who were engaging well with services tended to be ‘selected’ for referral by clinicians:

I got a sense of care coordinators wanting to put their best patients forward, like the ones that they were really confident in, in terms of engagement, yeah. Because it’s frustrating, because I felt like our trial was applicable to a lot more people than were put forward in the meetings. (Interview 4)

This is particularly problematic as the symptoms of psychosis can make engagement difficult and patients who did not have a good relationship with their care coordinator could have been unfairly excluded:

[A] lot of people who experience psychosis and are paranoid of other people may sometimes have not always had a great rapport with their care coordinator because they might have been wary of them, they might not have engaged much, and so potentially there was a bit of bias there from the clinician side of things in terms of who they had a better rapport with maybe. (Interview 7)

Another issue identified by the RAs was that referrals were influenced by clinicians’ personal views on what kind of patient was suitable for a particular trial. One RA noted a tendency for clinicians to refer young men into a particular trial because the digital intervention was thought to be particularly appealing to this group:

I think there was maybe stereotyping; maybe people were more likely to ask young men, compared to women […]. Maybe they… seeing a young man would, again like, trigger a thought in a clinician’s mind to tell them about the study. (Interview 15)

Although on the whole unwritten eligibility criteria were perceived by RAs as being applied spontaneously—and unhelpfully—by clinicians, sometimes research teams actively promoted a profile of the ‘ideal’ participant who would engage well. One RA said:

it’s not a criteria of the trial, but it is something that we would try and get across as if engagement was good in terms of sort of taking part in a research trial is what would be most ideal and beneficial. So I think we sort of pushed that for them to think about… (Interview 3)

Whilst practical considerations clearly need to be taken into account, such an approach could discourage clinical teams from referring eligible patients who are not well engaged with services and thus reduce the representativeness of the sample.

##### Relationships are the crux

There was a strong consensus that because clinical teams are such powerful players in trial recruitment, the key to successful trial recruitment was for research teams to establish and maintain positive relationships with individuals within clinical teams. As one RA put it:

It’s kind of people in positions of responsibility bearing you and the study in mind in the context of a really busy service. So having the team lead, a key psychiatrist, a key clin psych [clinical psychologist], if there’s a structure of the care coordination, having a key care coordinator holding the study in mind, giving regular little reminders, not just going to one presentation at the beginning, but actually booking them in monthly so that you’re a face that they know. And once you’re a face that they know, much more likely to come up to you and just say… (Interview 2)

RAs needed clinical teams to trust their professionalism in order to feel comfortable referring, which could be facilitated by having researchers working within clinical teams, as described here:

Something else that I think really helped with building that rapport was having a few people on our trial site team who were also embedded in the clinical teams because I think that really increased the trust between the project and the clinical staff, [….] I think that gave us a little more credibility potentially. (Interview 7)

Pre-existing relationships research team members had with clinical teams were also important. The PI’s close connection to a particular clinical team provided a fruitful source of referrals for the trial one RA was recruiting into:

There’s a community mental health team where we say we’re almost honorary members of staff, ‘cause our head PI [principal investigator] like worked there clinically for many years. So he has, like, quite good relationships with them, so they'll always give the time of day, and they’ve been like the highest referrers… (Interview 14)

#### Theme 4: NHS infrastructure factors

##### Systemic issues

RAs highlighted systemic issues affecting the NHS as a whole which undermined trial recruitment. They frequently acknowledged the strain the NHS was under and the resulting lack of time available to clinical teams to refer patients, for example:

[O]ne of the big sort of barriers was around clinicians’ time to think about the study and refer to the study and keep the study in mind. And I think it’s quite difficult because all of that is understandable in the context of the work environment and the realities of working in the NHS. So it almost feels like there needs to be change at a much higher level in terms of […] like people having, I guess, time for that within their role, like to support research. […] I guess overall you need more… there needs to be more funding (Interview 5)

As well as affecting numbers of referrals, systemic issues were also seen as impacting which patients received referrals. One RA had observed people from minority ethnic backgrounds being excluded from the opportunity to participate in trials due to marginalisation by clinical services:

I think white people are easier to recruit because of a whole host of institutional barriers that are in place for people with minority ethnic backgrounds […] You’re just massively under-recruiting for a whole host of reasons because people from those backgrounds just don’t have the same relationship with their care coordinator. We looked, and pretty much every single white person at one of the services had a care coordinator […] out of 300 and something people at the service, there were 70 people that didn’t have a care coordinator, and the majority of those people were black people. (Interview 13)

Another RA recognised that researchers could be complicit in the marginalisation of minority groups by focusing recruitment efforts on clinical services:

[I]t’s easiest, as a research team, to go into already established services. But one of the problems is that already established services might not have minority groups within them, so we’re kind of just, we’re feeding into that cycle and we’re just contributing to the problem. (Interview 15)

##### Alternative recruitment pathways

Whilst recruitment into trials involving patients with psychosis in the NHS traditionally requires teams to refer patients under their care, the RAs also described alternative pathways. These included community outreach, research registers, working with research delivery staff, including Clinical Research Network (CRN) RAs or clinical studies officers (CSOs), and self-referral. RAs viewed alternative recruitment pathways as a way of potentially improving the efficiency of recruitment processes and accessibility to patients:

[I]t’s being able to then like make connections with maybe more like ethnic minority community groups […] and maybe like generating more diverse self-referrals that way… (Interview 8)

[T]he research register is one that is quite effective because that is going towards the service user directly. And it’s usually our clinical studies officers, because they are like embedded within the research units of like the NHS Trust, so part of like research and delivery. And the CSOs usually have access to their research registers, and then they can call the service users directly because they have permission to do that. And then they can tell them all the studies that they have available that might sort of be in line with their sort of presenting difficulties or things that they have going on. (Interview 10)

However, increased accessibility could also mean increased work for research teams and more disappointed patients when they learn they are ineligible:

[Y]ou can put it [a study advert] on like Facebook, Twitter, whatever, but I think the only problem with that is you do get people who’ll want it, even though they won’t meet the criteria, so it’s going to give you a load of admin. (Interview 1)

One RA also described how CRN RAs could potentially undermine, rather than enable, patient agency by making decisions about the appropriateness of a particular study for a patient, rather than allowing patients to make these decisions themselves:

[T]hey [CRN RAs] were thinking about lots of other projects so then it kind of enabled, for lack of a better term, cherry-picking, for who would be suitable for one trial as opposed to offering lots of choice and being like, ‘here are all these projects running that actually you could be suitable for, what will best fit you?’ (Interview 12)

There is clearly a balance to be struck here. Some patients might find it overwhelming to be offered an extensive menu of trials, but the opportunity to choose from a smaller selection of trials which are likely to be suitable and of interest may be appropriate.

## Discussion

### Summary of findings

This study explored RAs’ experiences of recruiting patients with psychosis into clinical trials. The thematic analysis generated overarching themes representing four types of factors: patient, clinical team, research team, and NHS infrastructure—each with up to three subthemes. The findings indicate clear actions for promoting recruitment across multiple levels of the recruitment infrastructure.

The patient factors suggest patients are often keen to participate in trials, and the key to facilitating this is for the RAs to build trust. The clinical team factors indicate RAs see clinical teams as the most powerful stakeholders in trial recruitment, and therefore good relationships with them need to be established and actively maintained. The research team factors describe how RAs rely on their research teams for both practical and emotional support, and how the presence of lived experience expertise within the team is valuable for successful recruitment. The NHS infrastructure factors encapsulate the systemic issues within the NHS, which make recruitment challenging and for which there are no easy solutions, and also highlight potential additional recruitment pathways.

### Relationship to existing literature

That patients with psychosis want to take part in trials echoes what patients themselves have stated [26, 27], as does the co-existence of altruistic and personal motivations for participation [20, 27]. The willingness of patients to be randomised observed by the RAs here runs counter to previous research which found that patients with psychosis were unwilling to be randomised in a hypothetical psychological therapy trial [28]. Evidence of the importance of RAs succeeding in building trust with patients with psychosis in order to facilitate trial participation is novel, but chimes with the findings of a systematic review of trial recruitment and retention, which found that fostering trust may increase trial participation for patients with a range of health conditions [29]. In a related vein, Thong et al. [20] found that patients with FEP value having a rapport with trial researchers.

The NHS Constitution for England pledges to inform patients of research studies in which they are potentially eligible to participate [30] but the finding that the RAs did not see this on the trials they recruited into echoes what others have observed. Both empirical studies in the form of SWATs [15–19] and tacit learning by trialists recruiting patients with psychosis [31, 32] have reported clinical teams referring selectively or not at all. When clinicians select patients for referral based on criteria outside of the protocol, implicit bias may play a role, potentially exacerbating the marginalisation of already disadvantaged groups [33]. The finding that relationship management is an effective way to address gate-keeping is consistent with existing qualitative research [15, 17–19]. However, a systematic review of interventions to change the behaviour of clinicians recruiting into RCTs found that researchers simply increasing contact with clinical teams was insufficient, but a bespoke approach whereby a qualitative SWAT was conducted to pinpoint obstacles and develop solutions resulted in an increased proportion of eligible patients consenting [34]. That patients with psychosis from ethnic minority backgrounds can be marginalised by services thus curtailing opportunities to take part in research is important. It echoes the conclusions of a systematic review by Brown et al. [35], which found that the first barrier to patients from ethnic minority backgrounds participating in mental health research was the lack of opportunity due to reduced use of health services. People from black and ethnic minority backgrounds have higher rates of psychosis than white people [12] so it is critical that trial participation is made accessible to these groups. The need for equality, diversity, and inclusion to be governing principles in health care and research is increasingly recognised with initiatives such as NHS England’s Core20PLUS5 [36], the Health Research Authority and Medicines & Healthcare products Regulatory Agency’s Inclusion and Diversity Plan [37], and the National Institute for Health and Care Research’s INCLUDE project [38] seeking to reduce inequality and increase the involvement of under-served groups.

Whilst institutional knowledge may be a valuable resource, as the findings of this study indicate, research teams clearly need to be adaptable and open to new ways of working, particularly if inclusion is to be improved. The possibility of increased accessibility to trial participation via additional recruitment pathways complements the findings of a study by Iflaifel et al. [39], which explored mental health trial recruitment and recommended a mixture of online and offline methods tailored to the specific patient population. However, Iflaifel et al. [40] also suggest that online recruitment, which they define as social media advertisements, Google search engine advertisements, and website campaigns, may be unsuitable for patients with psychosis due to the reduced opportunity for emotional connection when compared with in-person recruitment. The RAs interviewed for this study did not report having recruited using online adverts targeted at patients. When they recruited remotely, the RAs continued to seek referrals from clinicians but interacted with clinicians and patients via video or telephone call, instead of face-to-face. It is evident that the RAs considered establishing a connection with patients to be of the utmost importance and remote methods were useful only when they contributed to, rather than undermined, this process.

With regard to specific alternative methods, recruitment via a research register has been endorsed by NHS patients with psychosis and clinical staff [41], and reported to be effective in a US schizophrenia research clinic [42]. Opt-out research registers are potentially more inclusive than opt-in [43]; however, this does not necessarily result in greater diversity in participants actually recruited into studies [44]. Other recruitment methods cited by the RAs, including self-referral, recruitment via community outreach, and the CRN, are under-researched in this patient group. Further research is also needed to explore the practicalities and identify whether the limitations voiced by RAs in this study can be mitigated.

The findings of this study indicate that lived experience input into trial recruitment is valuable, but it is unclear from existing research what the most effective ways of doing this are. Greenwood et al. [45] reported that there were qualitative improvements in recruiting patients with psychosis into their RCT when there was PPI involvement; for example, referrals were received from teams who had not previously referred. However, an RCT involving patients with severe mental health problems found that leaflets produced by PPI partners were not effective in increasing recruitment [46], which is consistent with the findings of Treweek et al. [47]’s systematic review of trial recruitment strategies.

### Recommendations for trialists

Recommendations resulting from this study include:

1. Train RAs in interpersonal skills and ensure sufficient time is allocated to both building trust with patients and establishing and maintaining relationships with clinical teams.
2. Recognising that the strain the NHS is under means that clinical teams have very limited time to invest in research, reduce the burden of referring for clinicians as far as possible, for example, by minimising paperwork.
3. Encourage clinicians to refer all patients who are potentially eligible, particularly from under-represented groups, to help ensure that trial participants are representative of those using services, and discourage RAs from seeking referrals of ‘ideal participants’ at the expense of recruiting inclusively.
4. Identify barriers to referring and misconceptions about patient eligibility by speaking directly to clinical teams, and develop targeted solutions to address these.
5. Ensure there is sufficient time and funding available to offer appointments in a range of locations and formats so patients are able to choose the method they are most comfortable with. This will inevitably incur additional expense, but enabling RAs to take the time to build trust with potential participants and travel to meet them at home where necessary is essential for the successful recruitment of this patient group.
6. Involve people with lived experience and from diverse backgrounds in the development and implementation of recruitment strategies.
7. Identify suitably qualified individuals to provide supervision and support RAs to manage the emotional challenges of recruitment and troubleshoot issues.
8. Use learning from previous trials to inform future recruitment practices where relevant.
9. Undertake community outreach activities with the aim of increasing diversity amongst trial participants and more accurately representing the target population.
10. A mix of recruitment strategies, including remote and alternative methods for example, self-referral and research registers, may help to increase accessibility to patients from under-represented groups, but these must be tailored to the target population. For example, it might be appropriate to invite self-referrals via a suitable social media platform for patients who are worried about leaving the house, whereas for patients experiencing paranoia digital methods may cause unease for a proportion.

### Strengths and limitations

To our knowledge, this is the first study of recruitment into clinical trials involving patients with psychosis, specifically focusing on the RA perspective. The RAs interviewed in this study had recruited into a number of different trials (although the majority of RAs only had personal experience of recruiting into a single trial) with more than half having recruited remotely, so one strength is that the findings capture a range of recruitment experiences, including methods beyond the traditional clinician referral route. However, only one of the RAs interviewed had experience working in a research delivery team, with the others all connected to specific trials, so there would be value in exploring the trial recruitment experiences of CRN RAs more extensively.

The RAs were recruited within a network of trialists working on trials of cognitive behavioural therapy-informed therapies within the NHS, which means the findings are most relevant to RCTs of this nature. Recruitment into trials of pharmacological interventions is likely to involve different challenges; for example, patients may have more reservations about taking part due to possible side-effects, and alternative recruitment pathways may be less appropriate due to the higher risks.

The trials the participating RAs had worked on were located in various regions of the UK, so there was some geographical diversity in the study. However, the RAs themselves were predominantly white British female graduates in their twenties. The lack of diversity is noteworthy and potentially a contributing factor to the challenge of recruiting patients from ethnic minority backgrounds. However, the study’s findings should be transferrable as the participants are likely representative of the current RA population. The gender imbalance seen in the participants in this study is also visible in Psychology students [48], and amongst practicing psychologists, where 84% are of White ethnicity and 80% are women [49].

Research teams may consider offering paid internships to students from under-represented groups as a way of increasing diversity amongst staff. This may in turn increase the diversity of participants recruited into trials. Research teams with experience of recruiting under-represented groups in the USA considered diversity in team members to be beneficial for recruitment because it meant they should reflect the participant population more closely [50].

The trial recruitment ecosystem described here is from the RAs’ perspective and would in all likelihood be seen differently by other stakeholders in the recruitment process. As the RAs interviewed perceived patients as generally keen to take part in trials and the most significant recruitment challenges originating with clinical teams, exploring the perspectives of these groups would be particularly valuable.

### Reflexivity

The first author worked as a trial coordinator on two trials that did not recruit to target, leading her to view recruitment as challenging, partly because clinical teams act as gatekeepers, and to be sympathetic to RAs’ struggles. The research team to which she belongs has a strong patient-centred ethos, which shaped the data collection and the analysis. The influence of patient-centred values is evident in her intention to explore the interactions between RAs and patients. It also had an impact on the way the interviews were conducted, for example, a reluctance to explore the risks encountered by RAs working with patients with psychosis due to a fear of perpetuating negative stereotypes about this population. Finally, the results clearly show the influence of such a stance, particularly the sub-themes ‘wanting to take part’ and ‘holding the power.’ However, these sub-themes are also grounded in the data.

The first author was both an insider and an outsider [51]. Being an ‘insider’ facilitated the recruitment of interviewees and meant she was familiar with the argot of clinical trials, so understood the jargon the RAs used. As well as having similar experience (and in some cases, a pre-existing professional relationship), the researcher also shared other characteristics with the participants—female gender and White British ethnicity (for the majority of the participants), and a degree in Psychology, which undoubtedly also contributed to the building of a rapport. However, due to her supervisory experience (which the RAs were aware of) some RAs seemed to perceive her as an ‘outsider’ who was monitoring their accounts for errors conducting trial procedures, which may have resulted in self-censoring.

## Conclusions

From the perspective of RAs, recruitment is a complex and multi-faceted process requiring careful judgement and influenced by many factors beyond their control. The findings of this study both cohered with and enriched existing findings on the recruitment of patients with psychosis into clinical trials. The findings have practical value for research teams and make it evident that in order to optimise trial recruitment, multiple strategies are needed. Building rapport both with patients and clinical teams should be prioritised for recruitment to be as efficient and inclusive as possible.

## Supplementary Information

Supplementary Material 1.

Supplementary Material 2.

## Data Availability

The full dataset (i.e. interview transcripts) is not available due to ethical and privacy restrictions. Participants provided consent for the publication of anonymous quotes only, with an agreement that entire transcripts would not be shared beyond the research team.

## Abbreviations

CRN: Clinical Research Network
CSO: Clinical studies officer
FEP: First episode psychosis
NHS: National Health Service
PI: Principal investigator
PPI: Patient and public involvement
RA: Research assistant
RCT: Randomised controlled trial
SWAT: Study within a trial
TA: Thematic analysis
