# Supplementary material for: Research assistants’ experiences recruiting patients with psychosis into clinical trials: a qualitative study
Source: Trials. 2025 May 30;26:180. doi: 10.1186/s13063-025-08882-y (PMC12124033; doi:10.1186/s13063-025-08882-y)
Supplement: Supplementary file 1 — Supplementary Material 1. [file 13063_2025_8882_MOESM1_ESM.docx]

# **Supporting Information**

## Topic guide development

The interviews were guided by a topic guide developed by the first author in collaboration with supervisors. It was informed by a review of the literature review and the first author’s experience working as a trial co-ordinator.

The interview covered six key topics (background, recruitment processes, liaison with clinical teams, liaison with patients, recruitment challenges, and ways of improving recruitment). There was an opportunity for the interviewee to provide any additional relevant information. Example questions for each topic are included below but only a selection of questions was asked to each participant. Probes e.g. ‘could you please tell me more about that?’ and ‘could you please give me an example?’ were used to seek further detail as needed.

The topic guide was piloted in the first two interviews and found to be suitable so with the permission of the participants, the data from these interviews was included in the analysis. The topic guide evolved as data collection progressed with questions on topics such as public and patient involvement (PPI) in recruitment strategies and the emotional aspects of recruitment added later.

## Topic guide

**Introduction:** *We are interested in hearing about how you have recruited patients with psychosis into clinical trials. This is so we can better understand the recruitment process and learn about which approaches tend to work well for this patient group and can be taken forward, and which aren’t so effective. There are no right or wrong answers and we aren’t evaluating you or your research team; we just want to learn from your experiences.*

1. **Background**

*Please could you tell me about your experience of recruiting patients with psychosis into clinical trial?*

- *What kinds of trials have you recruited participants into? Have you recruited participants with psychosis into any trials where the target population wasn’t specifically people with psychosis? If so, how did this differ?*
- *What training did you receive on recruitment? Was it useful?*

1. **Recruitment processes**

*Could you talk me through the typical process of recruitment from the outset to the point at which the patient consents?*

- *Do the process differ across trials (if interviewee has recruited into more than one) and if so, have you found any processes to be more effective than others?*
- *Do you have any experience of recruitment being changed processes during the course of the trial? If so, why were they changes made and what impact did these changes have?*
- *Have you recruited face-to-face and/or remotely? What impact does the recruitment method have?*
- *What roles do other research team members play in the recruitment process?*
- *Was any preparatory work undertaken e.g. pilot or feasibility study for the trials you have recruited into? If so, how did this inform the recruitment strategy?*

1. **Liaison with clinical teams**

- *How do you go about introducing a trial to a clinical team? What kinds of responses do you receive?*
- *What are the most effective ways of communicating with clinical teams? How do you build a rapport? How did you maintain the relationship throughout the trial?*
- *What kinds of questions do clinicians ask about trials? What are their concerns? What are they interested in finding out more about?*
- *How well do you think clinicians have understood the trials you’ve sought referrals into?*
- *How do you think clinicians decide whether or not to refer a particular patient?*
- *Are there some clinicians who refer more patients than others? Why do you think this is?*
- *What are the roles of those who have referred patients into trials e.g. care coordinator, psychiatrist? Have you noticed any differences across roles?*
- *How do you think clinical teams see their role in the recruitment process?*
- *Have you encountered any clinicians who were particularly supportive? What did they do that was helpful?*

1. **Liaison with patients**

- *How do you introduce a study to a patient who has been referred? What kinds of responses do you receive?*
- *How do you build a rapport with patients?*
- *What questions do patients ask about trials? What are they interested in finding out more about? What are their concerns? How do you address these concerns?*
- *How well do you think patients have understood the trials you’ve recruited into?*
- *How do you think patients decide whether to take part?*
- *What reasons do patients give for participating? What reasons do they give for declining?*
- *Have you encountered any patients who have changed their mind about taking part prior to/after consenting? If so, why was this?*
- *Do you have any experience of participants withdrawing/disengaging from the trial? Why do you think this was?*
- *Are there particular groups of people you have found it harder/easier to recruit? Why do think this was?*
- *Have you had any experience of patients referring themselves into trials? How did this go?*

1. **Recruitment challenges**

- *What challenges have you come across in the recruitment process e.g. in relation to clinical teams; patients?*
- *Did you discuss challenges with anyone else e.g. your supervisor, the wider research team?*
- *How did you deal with these challenges?*
- *What other potential solutions are there?*

1. **Ways of improving recruitment**

- *How do you think the process of recruiting patients with psychosis into clinical trials could be improved?*
- *How do you think research teams can facilitate referrals from clinical teams? Are there other ways patients could be reached?*
- *How do you think clinical trials can be made more accessible for patients with psychosis, particularly for groups who are under-represented?*
- *How do you think patients can be best supported to make an informed decision about whether to take part in a trial or not?*
- *What would you do differently in the future?*
- *What advice would you provide to other people recruiting into trials?*

1. **Additional information**

*Is there anything else relevant we haven’t covered that you would like to share?*

## Demonstrating rigour

The Consolidated Criteria for Reporting Qualitative Research (COREQ) (Tong et al., 2007) is a 32-item checklist designed to promote credibility in qualitative studies where data is collected by interview and focus groups was used to ensure the study was reported in a clear and rigorous manner. However, as Barbour (Barbour, 2001) observes, a completed checklist is not a guarantee of trustworthiness. Levitt et al. (2017) propose that trustworthiness depends instead on methodological integrity. The concept of methodological integrity is applicable to any qualitative design and refers to consistency between all aspects of the research (Levitt et al., 2017). When methodological integrity is achieved, the findings of the study are faithful to the data collected and the utility of the study in achieving the aims set out by the researchers is optimised (Levitt et al., 2017). In order to ensure methodological integrity in this study, care was taken to ensure the aims, philosophical underpinnings, participant characteristics, and methods of data collection and analysis were considered holistically and clearly articulated.

Reflexivity can also contribute to methodological integrity. As reflexive TA is not dependent on a single framework or theoretical standpoint, the decisions made by the researcher need to be interrogated (Braun & Clarke, 2022). Reflexivity helps to ensure rigour by shedding light on the ideas and assumptions which have led the researcher to the conclusions they have reached so others can consider their validity (Braun & Clarke, 2022). The researcher must not only be aware of these influences but also be able to articulate them, as Elliott, Fischer and Rennie, (1999) put it, ‘owning one’s perspective’ is a marker of quality. A reflexive log was therefore maintained throughout the study. The first author recorded their thoughts and feelings prior to undertaking the interviews, initial reflections immediately following each interview and the evolving analysis, particularly in response to discussions with supervisors. This was particularly important as the analysis was developed and the researcher’s conceptualisation of the study shifted, as described above.

In order to enhance the credibility of the analysis, a supervisor reviewed coding samples, as recommended by (Barbour, 2001). Rather than being a way to identify personal bias, this helped to foster reflexivity and to identify any oversights, for example encouraging the researcher to ask later participants about the emotional aspects of recruitment, which had been mentioned in early interviews (Braun & Clarke, 2022). Respondent validation is often considered to be an effective way of strengthening the credibility of a qualitative study’s findings but was not appropriate here (Mays & Pope, 2000). This is because the subjective and particular character of the findings is a strength, rather than a limitation, of reflexive TA (Braun & Clarke, 2022).

The transferability of the results – the extent to which the findings may be of value to others operating in different contexts and environments (Tracy, 2010) – was also considered. ‘Thick description’ was provided in order to situate the findings of this study and enhance their transferability (Polit & Beck, 2010). This description included the timeframe, context, and information about the participants: age, gender, ethnicity, and experience recruiting patients into clinical trials.

## Participant theme contributions

| **Theme** | **Participant** | | | | | | | | | | | | | | |
| --- | --- | --- | --- | --- | --- | --- | --- | --- | --- | --- | --- | --- | --- | --- | --- |
|  | 1 | 2 | 3 | 4 | 5 | 6 | 7 | 8 | 9 | 10 | 11 | 12 | 13 | 14 | 15 |
| ***Patient factors*** | | | | | | | | | | | | | | | |
| Wanting to take part | x |  | x |  | x | x | x | x | x | x | x | x | x |  | x |
| Building trust | x |  | x | x | x |  | x | x | x | x | x | x | x | x | x |
| ***Clinical team factors*** | | | | | | | | | | | | | | | |
| Holding the power | x | x | x | x | x | x | x | x |  | x | x | x | x | x | x |
| Suitability is a judgement | x | x | x | x | x | x | x |  |  |  | x | x | x | x | x |
| Relationships are the crux | x | x | x |  | x | x | x | x | x | x | x | x | x | x | x |
| ***Research team factors*** | | | | | | | | | | | | | | | |
| Lived experience expertise | x |  |  |  |  |  |  |  |  |  | x | x | x | x |  |
| Support system | x |  |  |  | x | x | x | x |  |  | x | x |  | x | x |
| Institutional knowledge | x |  | x |  | x | x | x |  |  | x |  | x |  | x |  |
| ***NHS infrastructure factors*** | | | | | | | | | | | | | | | |
| Systemic issues |  | x | x |  | x | x | x | x |  | x | x | x | x | x | x |
| Alternative recruitment pathways | x |  | x |  |  | x | x | x | x | x | x | x | x | x | x |

### References

Barbour, R. S. (2001). Checklists for improving rigour in qualitative research: a case of the tail wagging the dog? *BMJ*, *322*(7294), 1115-1117. <https://doi.org/10.1136/bmj.322.7294.1115>

Braun, V., & Clarke, V. (2022). *Thematic analysis : a practical guide*. SAGE Publications Ltd.

Elliott, R., Fischer, C. T., & Rennie, D. L. (1999). Evolving guidelines for publication of qualitative research studies in psychology and related fields. *Br J Clin Psychol*, *38*(3), 215-229. <https://doi.org/10.1348/014466599162782>

Levitt, H. M., Motulsky, S. L., Wertz, F. J., Morrow, S. L., & Ponterotto, J. G. (2017). Recommendations for designing and reviewing qualitative research in psychology: Promoting methodological integrity. *Qualitative Psychology*, *4*(1), 2–22. <https://doi.org/10.1037/qup0000082>

Mays, N., & Pope, C. (2000). Qualitative research in health care. Assessing quality in qualitative research. *BMJ*, *320*(7226), 50-52. <https://doi.org/10.1136/bmj.320.7226.50>

Polit, D. F., & Beck, C. T. (2010). Generalization in quantitative and qualitative research: myths and strategies. *Int J Nurs Stud*, *47*(11), 1451-1458. <https://doi.org/10.1016/j.ijnurstu.2010.06.004>

Tong, A., Sainsbury, P., & Craig, J. (2007). Consolidated criteria for reporting qualitative research (COREQ): a 32-item checklist for interviews and focus groups. *Int J Qual Health Care*, *19*(6), 349-357. <https://doi.org/10.1093/intqhc/mzm042>

Tracy, S. J. (2010). Qualitative Quality: Eight “Big-Tent” Criteria for Excellent Qualitative Research. *Qualitative inquiry*, *16*(10), 837-851. <https://doi.org/10.1177/1077800410383121>
