# Supplementary material for: Research assistants’ experiences recruiting patients with psychosis into clinical trials: a qualitative study
Source: Trials. 2025 May 30;26:180. doi: 10.1186/s13063-025-08882-y (PMC12124033; doi:10.1186/s13063-025-08882-y)
Supplement: Supplementary file 2 — Supplementary Material 2. [file 13063_2025_8882_MOESM2_ESM.docx]

## Completed COREQ checklist for study

Consolidated criteria for reporting qualitative studies (COREQ): 32-item checklist (Tong et al., 2007)

**Domain 1: Research team and reflexivity**

***Personal Characteristics***

1. Interviewer/facilitator

AB conducted all fifteen interviews as stated in the Methods section.

1. Credentials

The authors’ qualifications are stated on the title page.

1. Occupation

The authors held positions within universities and NHS trusts, as indicated by the affiliations on the title page.

1. Gender

AB is female as stated in the Reflexivity section.

1. Experience and training

AB had training and experience of qualitative interviewing as stated in the Procedure section.

***Relationship with participants***

1. Relationship established

In some cases, the interviewer had a pre-existing relationship with the interviewees, as discussed in the Ethical considerations section.

1. Participant knowledge of the interviewer

Participants were informed of the interviewer’s background and motivations for carrying out the study via the participant information sheet, as stated in the Participants section.

1. Interviewer characteristics

The interviewer’s characteristics are described in the Reflexivity section.

**Domain 2: study design**

***Theoretical framework***

1. Methodological orientation and Theory

Critical realist ontology and contextualist epistemology with reflexive TA as method of data analysis as stated in the Design and Data analysis sections.

***Participant selection***

1. Sampling

Purposive sampling was used with maximum variation sought in the sample, as stated in the Methods section.

1. Method of approach

A flyer was circulated within the researchers’ existing personal networks as stated in the Participants section.

1. Sample size

Sample size was decided based on the principle of ‘information power’ (Malterud et al., 2016) and data collection ceased at 15 participants, as stated in the Participants section.

1. Non-participation

The number of respondents who contacted the researcher expressing an interest in taking part but were not ultimately interviewed is stated with accompanying reasons in the Participant characteristics section.

***Setting***

| 1. Setting of data collection   Interviews were conducted remotely with the researcher either at home or in a private office as stated in the Procedure section.   1. Presence of non-participants   No one else was present as stated in the Procedure section.   1. Description of sample   Demographic characteristics of participants are described in the Participant characteristics section. |
| --- |

***Data collection***

1. Interview guide

A topic guide was used as stated in the Procedure section and is included in the supporting materials.

1. Repeat interviews

Participants were only interviewed once as stated in the Procedure section.

1. Audio/visual recording

Interviews were audio recorded as stated in the Procedure section.

1. Field notes

The researcher took field notes during the interviews and recorded reflections after each interview as stated in the Procedure section.

1. Duration

The range of interview durations is stated in the Participant characteristics section.

1. Data saturation

The concept of ‘information power’ (Malterud et al., 2016) is proposed as an alternative to saturation more in keeping with the study’s methodological orientation in the Participants section.

1. Transcripts returned

Transcripts were not returned to the participants for checking but were checked for accuracy by the researcher as stated in the Procedure section.

**Domain 3: analysis and findings**

***Data analysis***

1. Number of data coders

The data was coded by one researcher as stated in the Data analysis section.

1. Description of the coding tree

Coding followed the process described by Braun and Clarke (Braun & Clarke, 2022) as described in the Data analysis section.

1. Derivation of themes

The themes were derived inductively as stated in the Data analysis section.

1. Software

NVivo was used to code the majority of the transcripts as stated in the Data analysis section.

1. Participant checking

Participant checking was not used, which is consistent with the principles of reflexive TA (Braun & Clarke, 2022).

***Reporting***

1. Quotations presented

All themes included in the Themes section are supported by quotations from the interviews with each interviewee quoted at least twice and quotations identified by a participant number.

1. Data and findings consistent

The interpretation of the findings is considered in the Discussion.

1. Clarity of major themes

The major themes are clearly stated in the Results section.

1. Clarity of minor themes in the Themes section.

The sub-themes (minor) themes are stated in the Themes section.

**References**

Braun, V., & Clarke, V. (2022). *Thematic analysis : a practical guide*. SAGE Publications Ltd.

Malterud, K., Siersma, V. D., & Guassora, A. D. (2016). Sample Size in Qualitative Interview Studies: Guided by Information Power. *Qualitative Health Research*, *26*(13), 1753-1760. <https://doi.org/10.1177/1049732315617444>

Tong, A., Sainsbury, P., & Craig, J. (2007). Consolidated criteria for reporting qualitative research (COREQ): a 32-item checklist for interviews and focus groups. *Int J Qual Health Care*, *19*(6), 349-357. <https://doi.org/10.1093/intqhc/mzm042>
